# Impact of Design Variations and Infill Density in 3D-Printed PLA Components

**DOI:** 10.3390/polym17243336

**Published:** 2025-12-18

**Authors:** Pradeep Raja, Karthik Babu, Elif Kaynak, Oisik Das

**Affiliations:** 1 School of Marine Engineering and Technology, Indian Maritime University, Kolkata Campus, P - 19, Taratalla Road, Kolkata 700 088, India; 2 Department of Mechanical Engineering, Rajiv Gandhi Institute of Petroleum Technology, Sivasagar Campus, Assam 785697, India; kbabu@rgipt.ac.in; 3 Department of Civil, Environmental and Natural Resources Engineering, Luleå University of Technology, 97187 Luleå, Sweden; elif.kaynak@associated.ltu.se

**Keywords:** 3D printing, polylactic acid, lightweight structures, structural design optimization, finite element analysis (FEA)

## Abstract

3D printing offers the ability to fabricate lightweight structural profiles with controlled infill and geometry. This study examines the mechanical behaviour of 3D-printed polylactic acid (PLA) structures with a 10% infill density under four load conditions (10, 15, 20, and 25 N). Four designs (M1, M2, M3, and M4), representing commonly used structural profiles found in beam and column applications, were analysed using ANSYS finite element simulations. Each design was evaluated under roller and nodal boundary conditions to study deformation, stress, and strain responses. Three-point flexural tests were also carried out on all four designs, and the measured peak flexural stress and apparent flexural modulus were compared with the simulated stiffness values. Both the simulations and experimental results showed that Design M3 exhibited the highest stiffness and more consistent behaviour compared to the other designs, while Design M4 showed higher deformation and lower bending resistance. Roller supports generally reduced deformation through better load distribution, whereas nodal supports increased local stiffness in selected designs. Although the magnitude of stiffness differed between simulation and experiment, the ranking of the designs remained consistent. Overall, the study confirms that the geometry plays an important role in their load-bearing performance, and the numerical model provides a reliable tool for comparing and selecting suitable designs before fabrication.

## 1. Introduction

The advent of advanced manufacturing processes, particularly 3D printing, has influenced multiple industries by enabling the creation of complex structures with enhanced design flexibility, cost efficiency, and sustainability [1,2]. 3D printing, also known as additive manufacturing, allows precise fabrication of components layer by layer, reducing material waste and enabling the production of highly customised parts [3,4]. In construction, companies like ICON have utilised 3D printing to build structurally stable and sustainable homes, such as those in the Community First! Village in Texas and disaster-resistant housing in Mexico and Haiti using its Vulcan printer. These innovations highlight the potential of 3D printing in addressing housing challenges with speed, affordability, and reduced environmental impact [5,6]. Similarly, in aerospace, companies like Relativity Space fabricate over 85% of their rockets using 3D printing, offering lighter and more efficient designs [7,8]. In structural applications, 3D printing has demonstrated significant potential. For example, the MX3D bridge in Amsterdam shows the integration of robotic 3D printing and metal materials to construct a structurally functional and sustainable pedestrian bridge. Similarly, the Office of the Future in Dubai is a fully functional 3D-printed office building, illustrating how additive manufacturing can be applied to large-scale infrastructure projects [9,10]. These examples highlight how 3D printing supports the creation of functional structures while promoting sustainability through efficient material use. A key material for 3D printing is PLA (Polylactic Acid), a biodegradable polymer derived from renewable resources. PLA is widely used due to its low carbon footprint and compatibility with sustainable practices [11,12]. Its adoption in structural applications aligns with global efforts to reduce environmental impact [13,14]. These characteristics make PLA suitable for applications ranging from rapid prototyping to end-use structural components [4,15]. Moreover, PLA has been successfully incorporated into concrete reinforcement, improving its tensile strength and demonstrating its potential for hybrid applications in structural engineering [16,17].

Additionally, 3D-printed materials are increasingly used in load-bearing structural applications, where deformation resistance and stress management are critical. For instance, PLA has been employed in medical scaffolds for tissue engineering and aerospace components requiring lightweight yet durable structures [10,11]. Despite its growing adoption, there remains a research gap in understanding the behaviour of 3D-printed materials under varying support configurations, such as roller and nodal designs. Roller support provides uniform load distribution, minimising deformation and stress concentration, whereas nodal supports concentrate stress at fixed points, affecting structural stability [12]. Evaluating these configurations is essential to optimise the mechanical performance of load-bearing designs [13].

Recent studies have also examined the mechanical and tribological performance of 3D-printed PLA under various loading and surface interaction conditions. Findings show that infill density, printing orientation, and layer bonding strongly influence tensile, flexural, and impact behaviour, while surface roughness and contact conditions affect wear resistance and frictional response [18,19]. These studies highlight the importance of understanding both the material behaviour and geometric design when evaluating the structural performance of PLA components. This study evaluates 3D-printed PLA components in roller and nodal configurations to optimise their structural performance underload. Simulations allow for the assessment of structural integrity under varying conditions, leading to optimised designs ready for real-world applications, such as in lightweight structural components for the automotive, aerospace, and marine industries, where performance, durability, and material efficiency are crucial [14]. In this study, finite element simulations were performed to analyse four different structural designs (M1 to M4) under two support conditions, roller and nodal. The models used PLA material with a 10 percent infill density, and the analysis focused on examining their deformation, stress, and strain responses.

## 2. Materials and Methods

### 2.1. Materials

The material used in this study was polylactic acid (PLA), a biodegradable thermoplastic derived from renewable resources, chosen for its favourable mechanical properties, ease of processing, and environmental benefits. The specific grade of PLA used, supplied by 3idea Technologies, had properties including a density of 1.25 g/cm^3^, tensile strength of 60 MPa, Young’s modulus of 3.5 GPa, and elongation at break of 6%.

### 2.2. 3D Printing Parameters

A 10% infill density was selected based on preliminary trials and insights from previous studies, with the aim of achieving an optimal balance between material efficiency and structural performance. A low infill density significantly helps reduce material usage and printing time, while ensuring adequate load-bearing capacity, aligning with the goals of sustainable and cost-effective production. The Gyroid infill pattern was used due to its uniform stress distribution and isotropic mechanical behaviour, which enhances structural reliability even at lower densities. The infill orientation was maintained at 45°, a common practice to promote even load distribution during mechanical testing.

**Figure 1:**
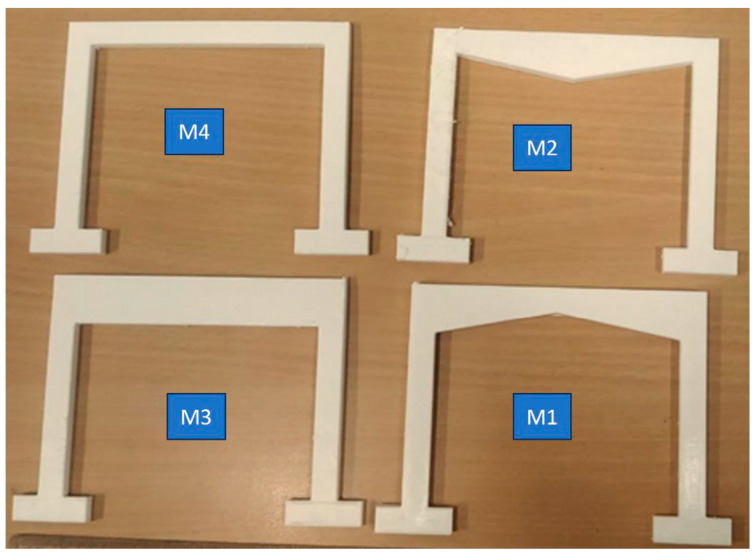
3D printed PLA specimens with varying designs and uniform cross-section.

Four distinct structural designs (M1, M2, M3, and M4) were modelled using AutoCAD software (version 24.1) and analysed under roller and nodal support configurations to evaluate their mechanical performance. While conventional load-bearing beams are typically rectangular, this study explores alternative geometries by varying the cross-sectional shape and area of the beam elements. As shown in Figure 1, each design reflects a unique profile, M1 features a shallow arch, M2 a tapered inverted V, M3 a uniform rectangular profile, and M4 a wide flanged (I section like) configuration. These variations were intentionally designed to study how changes in geometry affect structural behaviour, and to identify the optimised load bearing design through finite element analysis. The designs were fabricated using a MakerBot 3D printer with settings including a layer height of 0.2 mm, nozzle diameter of 0.4 mm, printing speed of 60 mm/s, infill density of 10%, printing temperature of 210 °C, and bed temperature of 60 °C. The printed specimens are presented in Figure 1.

### 2.3. Numerical Simulation Parameters

Finite element analysis (FEA) was conducted using ANSYS Workbench (ANSYS 2024 R2) software to simulate the mechanical behaviour of each design under various external loads, primarily compressive and contact loads, to replicate the operational conditions experienced by roller and nodal components. Static structural analysis was performed with convergence criteria set to ensure less than 5% error.

A mesh refinement study was performed, and the mesh density was increased until the variation in maximum deformation and von Mises stress between successive refinements was below 5%. The converged mesh obtained from this criterion was used for all simulations. The FEA setup included importing CAD models into ANSYS Workbench, where default meshing settings generated tetrahedral elements with an automatically determined element size based on model geometry. An isotropic material model was used because all specimens were printed with identical parameters and low infill, where the effective response is dominated by the cellular structure and provides a consistent basis for comparing geometric designs.

PLA material properties, including Young’s modulus, Poisson’s ratio, and density, were assigned to the model. Boundary conditions were set for roller configurations, with one end of the specimen fixed and the other end allowed to roll, simulating a simply supported beam, and for nodal configurations, with fixed supports applied at specified nodes to mimic realistic support conditions. Load cases of 10, 15, 20, and 25 N were applied to the models, distributed uniformly across the surface area of the specimen. A load range of 10–25 N was selected to keep the material response within the expected linear elastic region of PLA under small deflection conditions.

### 2.4. Three-Point Flexural Testing

Three-point flexural tests were conducted to evaluate the bending performance of the four 3D-printed PLA designs (M1-M4). A universal testing machine was used with a constant crosshead displacement rate of 2 mm/min, and load–displacement data were recorded throughout each test. All specimens were tested using the same loading configuration and test parameters to ensure consistency. The apparent flexural modulus was calculated from the initial linear region of the load response, while the peak flexural stress was obtained from the maximum load sustained prior to failure. These values were used to compare the relative structural behaviour of the different geometries.

## 3. Result and Discussion

The FEA performed on the four PLA designs (M1, M2, M3, and M4) under varying loads provided a comprehensive insight into their mechanical behaviours, specifically in terms of deformation, stress, and strain. The stress–strain behaviour of all sample designs is presented subsequently, which highlights the differences in mechanical response between the roller and nodal configurations under applied load.

### 3.1. Deformation Analysis:

**Figure 2:**
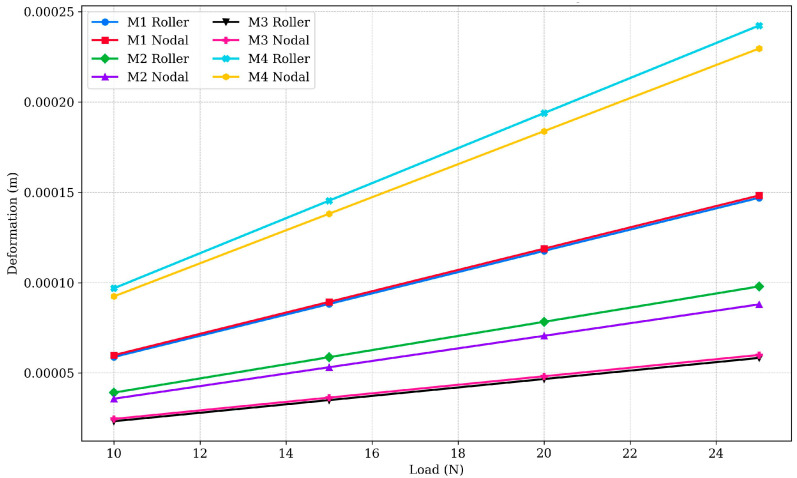
Deformation vs. load for PLA designs M1 to M4 under roller and nodal support conditions.

**Figure 3:**
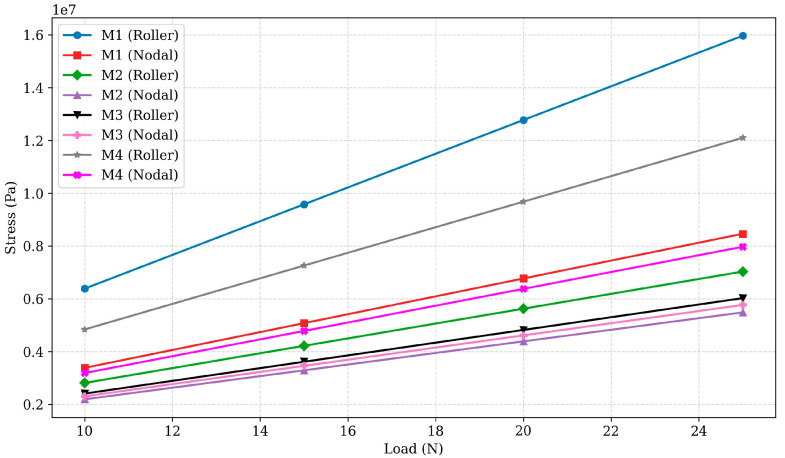
Stress vs. load for PLA designs M1 to M4 under roller and nodal support conditions.

Deformation analysis of the designed structural member is an important parameter in determining the stability and structural integrity of a material. The deformation data for the different designs under varying loads are presented in Figure 2 and Figure 3. For Design M1, deformation in the roller configuration ranged from 5.8974 × 10^−5^ m at 10 N to 1.47 × 10^−4^ m at 25 N, while the nodal configuration ranged from 5.8909 × 10^−5^ m to 1.4727 × 10^−4^ m. The percentage difference between these configurations was minimal (less than 0.5%), indicating nearly identical performance and suggesting that both configurations are equally effective in resisting deformation under load. This suggests that the roller design’s ability to more evenly distribute the load may result in slightly reduced deformation compared to the nodal design, although the difference is negligible. Design M2 displayed intermediate deformation characteristics, with roller configuration values ranging from 3.9172 × 10^−5^ m at 10 N to 9.7931 × 10^−5^ m at 25 N, while the nodal configuration ranged from 3.4799 × 10^−5^ m to 8.6998 × 10^−5^ m. The roller configuration exhibited approximately 10–12% lower deformation than the nodal configuration, suggesting that the roller design provides slightly better performance in minimizing deformation due to more effective load distribution.

Design M3 was the most stable among all the tested designs, exhibiting the lowest deformation values. The roller configuration ranged from 2.3342 × 10^−5^ m at 10 N to 5.8355 × 10^−5^ m at 25 N, while the nodal configuration ranged from 2.3601 × 10^−5^ m to 5.9003 × 10^−5^ m. The percentage variation between configurations was minimal (less than 2%), indicating consistent deformation resistance in both designs. This improved performance is attributed to M3’s optimised geometry, which enhances its ability to resist deformation under varying loads and contributes to its structural stability. In contrast, Design M4 exhibited the highest deformation values among all the configurations. The roller configuration ranged from 9.6922 × 10^−5^ m at 10 N to 2.423 × 10^−4^ m at 25 N, while the nodal configuration ranged from 9.1426 × 10^−5^ m to 2.2857 × 10^−4^ m. The roller configuration exhibited approximately 5–8% higher deformation than the nodal configuration, indicating less effective load distribution. These results suggest that Design M4 may be less suitable for applications requiring high structural integrity, as its design leads to greater displacement under load.

From the deformation analysis results, it can be observed that nodal configurations generally undergo higher deformation compared to their roller counterparts due to less effective load distribution. However, Design M3 (both roller and nodal) demonstrated better performance in minimising deformation, with minimal variation between configurations, indicating higher structural integrity and stability. The consistently lower deformation in roller configurations can be attributed to their ability to absorb and dissipate energy more effectively, reducing stress concentrations and overall displacement. Despite all samples being printed with the same infill density of 10%, Design M3’s optimised geometric design enhances load distribution, making it the most resilient design under the varying loads.

**Figure 4:**
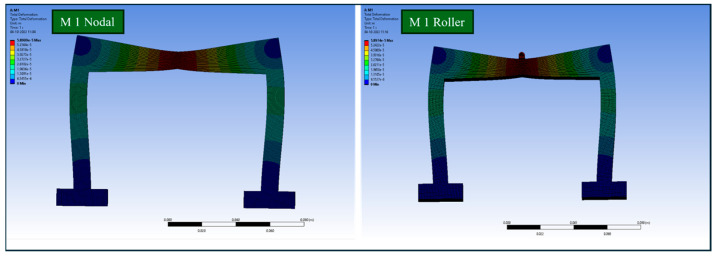
Total deformation of M1 specimens in roller and nodal configurations under a compressive load of 10 N.

**Figure 5:**
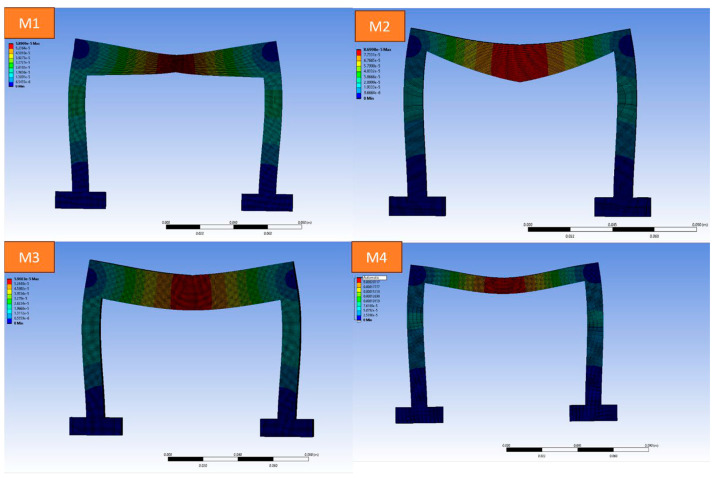
Total deformation of all design specimens in nodal configuration.

Figure 4 compares the total deformation of M1 specimens under nodal and roller configurations, demonstrating minimal differences between the two designs, indicating comparable deformation resistance. Figure 5 highlights the total deformation of all design specimens, revealing that Design M3 exhibited the lowest deformation, showcasing better structural stability, while Design M4 displayed the highest deformation, requiring further optimisation.

### 3.2. Stress Analysis

Stress analysis was carried out to determine the load-bearing capacity and potential failure points when the different design specimens were subjected to external loads. The von Mises stress criterion was used to assess material failure, comparing the maximum induced stresses against the known tensile strength of PLA to evaluate structural integrity. The variation in stress with load for all designs is presented in Figure 3.

For Design M1, the roller configuration exhibited stress values ranging from 6.3854 × 10^6^ Pa at 10 N to 1.5963 × 10^7^ Pa at 25 N, while the nodal configuration ranged from 3.385 × 10^6^ Pa to 8.4624 × 10^6^ Pa. The roller configuration showed approximately 90% higher stress, highlighting the nodal design’s more effective stress management. However, the significant stress variation suggests that M1 may not be suitable for applications requiring uniform stress distribution across configurations. For Design M2, the roller configuration stress values ranged from 2.8125 × 10^6^ Pa to 7.0312 × 10^6^ Pa, while the nodal configuration ranged from 2.194 × 10^6^ Pa to 5.485 × 10^6^ Pa. The roller configuration exhibited approximately 28% higher stress, showing that the nodal configuration handles stress more effectively. This makes M2 a balanced design for moderate-load applications, though the roller configuration could benefit from better load distribution.

For Design M3, the roller configuration showed stress values ranging from 2.41 × 10^6^ Pa to 6.025 × 10^6^ Pa, while the nodal configuration ranged from 2.3071 × 10^6^ Pa to 5.7677 × 10^6^ Pa. The variation between the two configurations was approximately 4.5%, indicating that both configurations effectively manage stress. The minimal variation highlights M3’s ability to uniformly distribute stress, making it useful and suitable for high-load applications. Its optimised geometry minimises stress concentrations, enhancing structural resilience and reducing the risk of material failure under load. For Design M4, the roller configuration exhibited stress values ranging from 4.8391 × 10^6^ Pa to 1.2098 × 10^7^ Pa, while the nodal configuration ranged from 3.1882 × 10^6^ Pa to 7.9706 × 10^6^ Pa. The roller configuration showed 30–40% higher stress, indicating poor load distribution and susceptibility to stress concentration. These results suggest that M4 may require geometric optimisation to reduce stress levels and improve structural performance under high loads.

The stress analysis highlights that Design M3 (Nodal) exhibited the lowest stress values among all configurations, suggesting it is the most suitable design for applications requiring high load-bearing capacity with minimal stress. While other nodal configurations, such as Designs M1 and M4, exhibit higher stress levels, M3 demonstrates effective stress management, making it an ideal design under loading conditions.

### 3.3. Strain Analysis

**Figure 6:**
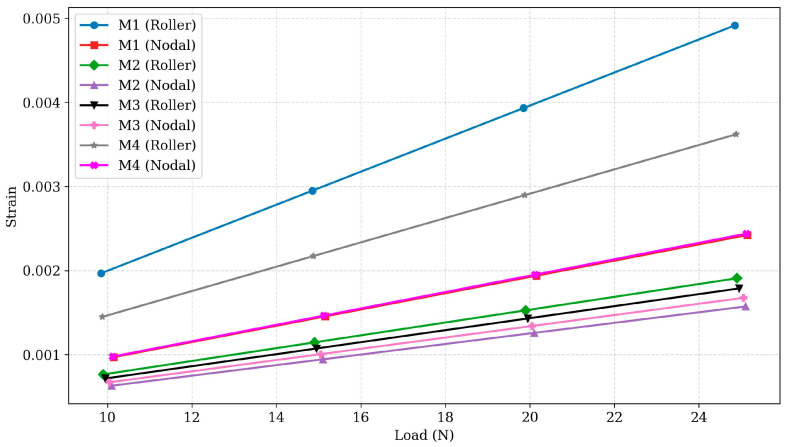
Strain vs. load for PLA designs M1 to M4 under roller and nodal support conditions.

**Figure 7:**
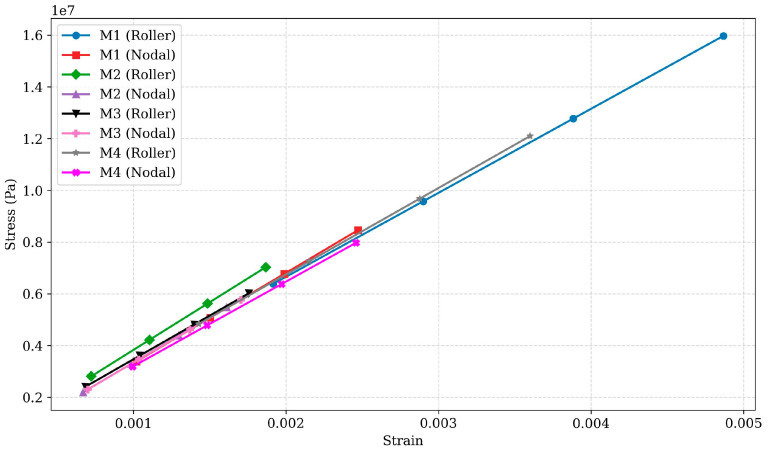
Stress vs. strain for PLA designs M1 to M4 under roller and nodal support conditions.

Strain analysis of the designs is essential for understanding their elastic behaviour and resilience. The strain data for different designs are presented in Figure 6 and Figure 7. For Design M1, the roller configuration exhibited strain values ranging from 0.0019664 at 10 N to 0.0049161 at 25 N, while the nodal configuration ranged from 0.0009689 to 0.0024223. The roller configuration had approximately 100–103% higher strain than the nodal configuration, indicating a reduced elastic recovery in the roller design. Despite similar deformation values, the roller configuration exhibited significantly higher stress and strain, reflecting less efficient load distribution. In contrast, the nodal configuration managed stress and strain more effectively, highlighting its efficient performance for applications requiring consistent structural integrity. Design M2 exhibited strain values ranging from 0.00076298 to 0.0019075 in the roller configuration and from 0.00062873 to 0.0015718 in the nodal configuration. The roller configuration showed approximately 20–22% higher strain, reflecting reduced elastic recovery compared to the nodal configuration. While the roller configuration exhibited 10–12% lower deformation, its higher strain values highlight its reduced efficiency in handling elastic distortion.

However, in contrast, the nodal configuration effectively minimises both stress and strain, making it more suitable for applications requiring resilience and consistent performance under loading conditions. Design M3 exhibited the lowest strain values among all designs, with the roller configuration ranging from 0.00071464 to 0.0017866, and the nodal configuration ranging from 0.00066971 to 0.0016743. The percentage variation between configurations was approximately 6.7%, confirming M3’s enhanced elasticity and resistance to deformation. Combined with its low stress (variation ~ 4.5%) and deformation (variation < 2%), Design M3 demonstrates strong structural integrity and consistent performance under load.

These characteristics make it the most reliable choice for applications requiring high durability and minimal deformation. Design M4 exhibited the highest strain values in its roller configuration, ranging from 0.0014482 to 0.0036205, while the nodal configuration ranged from 0.00097528 to 0.0024382. The roller configuration had approximately 48.5% higher strain than the nodal configuration, reflecting greater susceptibility to elastic deformation. Combined with its high stress and deformation values, the roller configuration demonstrates limited efficiency in bearing load.

The strain analysis indicates that nodal configurations generally exhibit lower strain compared to roller designs, indicating better resilience and deformation resistance under stress, as shown in Figure 5. This trend is evident in Designs M1, M2, and M4, where nodal configurations exhibit 20–100% lower strain, enhancing their suitability for applications requiring high durability. In contrast, Design M3 exhibits minimal strain differences (approximately 6.7%) between configurations, highlighting its consistent load-handling capability and resilience across both configurations.

High strain values in the roller configuration of Design M4 (0.0014482 to 0.0036205) suggest significant deformation under loading conditions, which might compromise its structural integrity. This also highlights the importance of optimising material distribution and load paths to reduce excessive deformation. Similarly, the consistently low strain values in both configurations of Design M3 demonstrate its better performance in terms of resilience and deformation resistance, making it a suitable choice for applications where maintaining structural integrity is critical.

### 3.4. Comparative Analysis of Numerical and Experimental Results

A detailed comparison between the three-point flexural experiments and the finite element simulations was conducted to evaluate how accurately the numerical model replicates the mechanical behaviour of the four 3D-printed PLA geometries. From the FEA, the effective bending stiffness of each design was obtained by performing a linear fit on the load–deformation response between 10 and 25 N. The resulting stiffness values were approximately 428.4 N/mm for M3 (Roller), 255.3 N/mm for M2 (Roller), 170.2 N/mm for M1 (Roller), and 103.2 N/mm for M4 (Roller), with nodal configurations exhibiting the same performance hierarchy. These values confirmed the numerical stiffness order of M3 > M2 > M1 > M4, consistent with the deformation and stress distribution patterns described in earlier sections of the manuscript.

**Figure 8:**
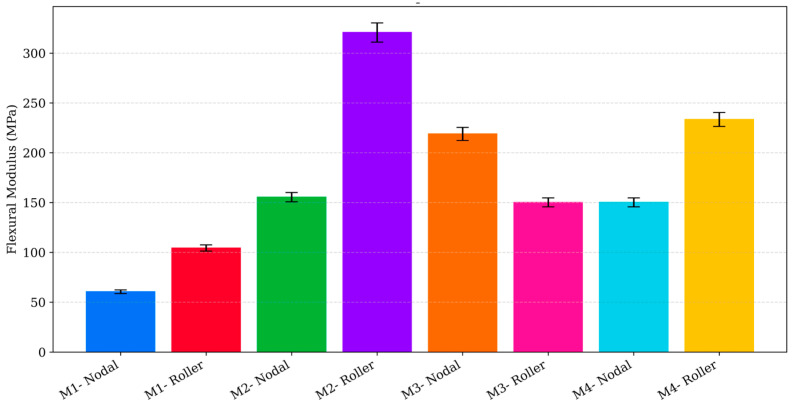
Experimental flexural modulus of 3D-printed PLA designs (M1 to M4) under three-point bending.

**Figure 9:**
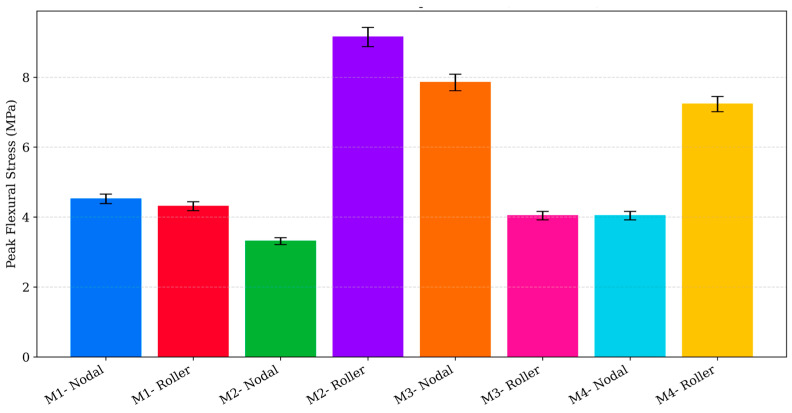
Peak flexural stress of 3D-printed PLA designs (M1 to M4) under three-point bending.

Experimentally, the apparent flexural modulus for each design was determined from the initial linear region of the three-point bending load response. Due to the cellular and heterogeneous characteristics of low-infill 3D-printed PLA, the experimentally measured modulus values occupied a different absolute scale compared to the FE predictions. The experimentally obtained flexural modulus and peak flexural stress values for all designs are summarised in Figure 8 and Figure 9. The apparent moduli ranged from 94.6 to 100.2 MPa for M1, 129.8–326.0 MPa for M2, 139.3–212.1 MPa for M3, and 139.3–225.4 MPa for M4. When normalised relative to M3 (Roller) to allow direct comparison of relative stiffness trends, the experimental stiffness ratios were 0.72 (M1-Roller), 2.34 (M2-Roller), 1.52 (M3-Nodal), and 1.62 (M4-Roller). In contrast, the FE-derived relative stiffness ratios were 0.40 (M1-Roller), 0.60 (M2-Roller), 0.99 (M3-Nodal), and 0.24 (M4-Roller). The deviation between numerical and experimental stiffness ratios therefore ranged from approximately 28% to 85%, with M2 (Roller) and M4 (Roller) exhibiting the largest overestimation of stiffness in the experimental measurements.

Despite these quantitative discrepancies, both simulation and experiment show clear directional agreement. In every case, M3 consistently appears among the stiffest designs, while M4 demonstrates the lowest resistance to bending, reflecting the influence of geometry on load transfer and structural efficiency. The differences in absolute stiffness values arise because the FE model assumes a homogeneous, isotropic PLA material, whereas the experimental specimens behave as low-infill cellular structures whose mechanical response is strongly affected by filament path continuity, internal porosity, and geometric transitions. Additionally, the experimentally derived apparent modulus inherently incorporates the structural moment of inertia and the local stress effects associated with each nonstandard cross section. These influences are not captured in the linear elastic material model used in the finite element analysis.

Overall, while the FE model does not replicate the exact magnitudes of experimental stiffness, it accurately predicts the relative performance hierarchy governed by geometry. This validates the simulation framework for comparative structural assessment of low-infill PLA components and demonstrates that specimen geometry plays an important role in determining bending stiffness and load-carrying capability. Both the FEA and the experimental flexural tests showed linear stress–strain behaviour within this range, confirming that all designs remained within the elastic region. The combined results confirm that numerical simulation is a reliable tool for ranking design performance, even though quantitative calibration would be required for precise stiffness prediction.

## 4. Conclusions

The investigation of four 3D-printed PLA geometries under roller and nodal loading conditions demonstrated a clear influence of structural design on deformation behaviour, stiffness, and overall load-bearing capability. Numerical simulations showed that Designs M1 and M3 exhibited lower deformation and more favourable stress distribution compared to M2 and M4, with roller configurations generally providing improved load dispersion. Among all designs, M3 consistently displayed the highest stiffness and the most stable deformation response, whereas M4 exhibited pronounced stress concentration and reduced structural integrity.

The experimental three-point flexural tests supported these numerical observations. The measured peak flexural stresses and apparent moduli confirmed the performance hierarchy predicted by the FE model, with M3 achieving the greatest bending resistance and M4 the lowest. Although absolute stiffness values differed between simulation and experiment due to the cellular nature of low infill printed structures, the relative performance trends remained consistent across both methods. This agreement validates the suitability of the FE framework for comparative structural assessment of complex 3D-printed geometries.

Overall, the combined numerical and experimental evaluation confirms that geometric design plays an important role in the mechanical performance of low-infill PLA components. Among the tested configurations, M3 showed the highest stiffness and lowest deformation among the tested designs for applications requiring enhanced stiffness and reduced deformation, whereas M4 would benefit from further optimisation to improve load distribution and bending stability.

## Data Availability

The original contributions presented in this study are included in the article. Further inquiries can be directed to the corresponding authors.
